# Successful management of severe hepatotoxicity induced by combined chemotherapy and immune checkpoint inhibitor: a case report

**DOI:** 10.3389/fmed.2026.1886401

**Published:** 2026-08-12

**Authors:** Minjie Jiang, Feifei Li, Dezhao Li

**Affiliations:** Department of Infectious Disease, Shandong Provincial Hospital Affiliated to Shandong First Medical University, Jinan, China

**Keywords:** case report, chemo-immunotherapy, favorable prognosis, hepatic impairment, immune-related adverse

## Abstract

With the increasing use of immune checkpoint inhibitors (ICIs), acute hepatitis as an immune-related adverse event (irAE) is expected to rise, particularly with the growing use of combination regimens with chemotherapy. However, the hepatotoxic risk of this strategy remains unclear. We report a 47-year-old woman with breast cancer and no history of chronic liver disease who developed severe hepatic impairment after fewer than two cycles of combined chemotherapy and immunotherapy. The patient was successfully treated with immunosuppressive therapy and an artificial liver support system (ALSS). This case highlights the importance of close monitoring in patients receiving combined chemo-immunotherapy and early intervention for suspected immune-mediated liver injury.

## Introduction

ICIs have emerged as a major breakthrough in cancer therapy and are now considered a key treatment modality alongside surgery, radiotherapy, and chemotherapy. Combination regimens, such as paclitaxel with programmed cell death protein-1 (PD-1) blockade, have shown promising efficacy in breast cancer (1). However, immune-related hepatitis is a recognized complication of ICI therapy, particularly when combined with chemotherapy. Most cases are mild and self-limiting (2), while severe hepatic impairment remains extremely rare (3). A meta-analysis by Guo et al. (4) reported a higher incidence of hepatitis of any grade in patients receiving PD-1/PD-L1 inhibitors combined with chemotherapy compared with chemotherapy alone, with rare treatment-related deaths due to severe hepatotoxicity. Here, we report a case of early onset hepatitis following combined chemotherapy and immunotherapy, successfully managed with corticosteroids and artificial liver support, highlighting the importance of early recognition and intensive management in severe cases.

## Case presentation

**Figure 1:**
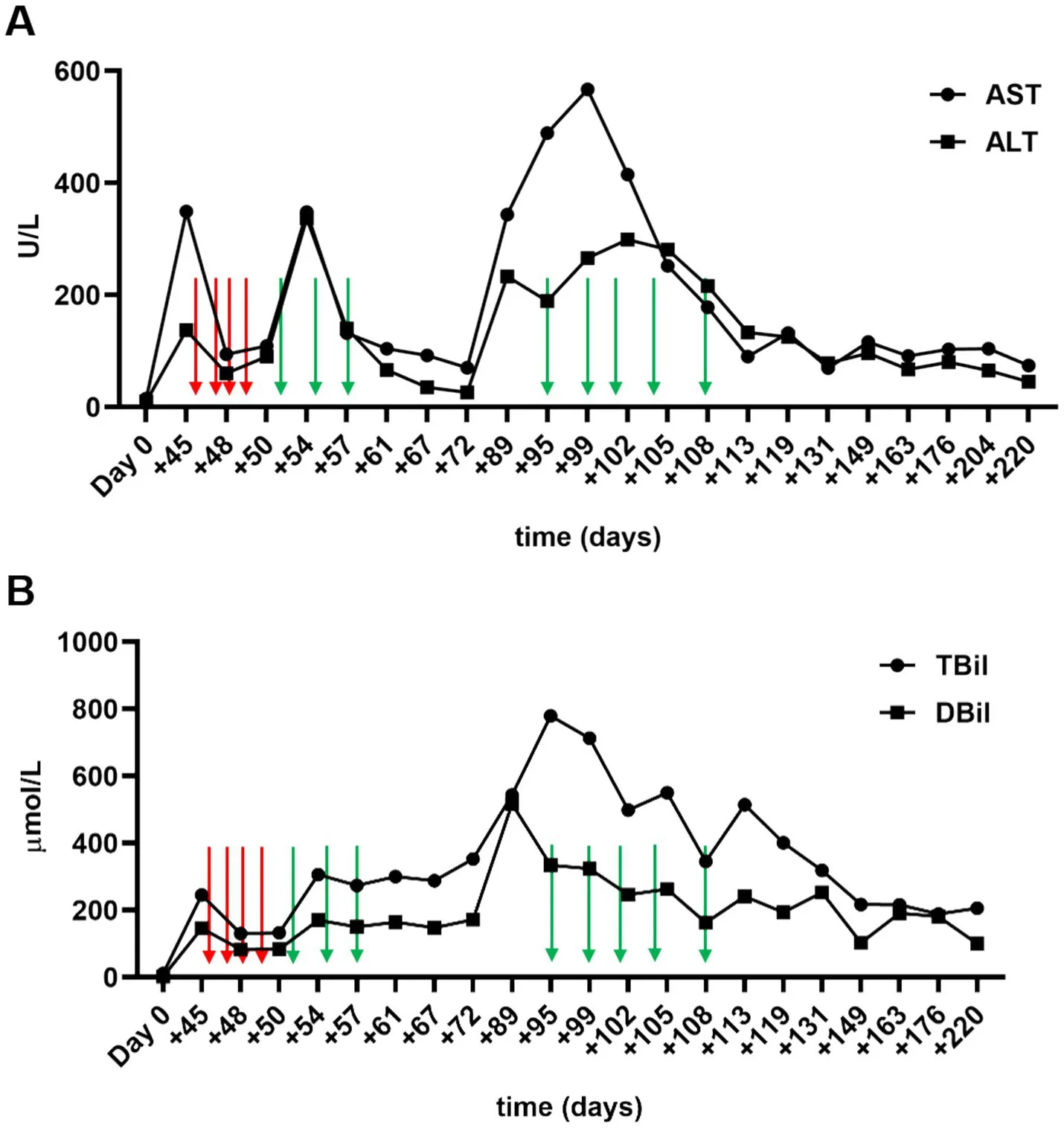
Laboratory data of this patient, obtained during hospitalization. **(A)** AST, ALT; **(B)** TBil, DBil. Red arrows indicate PE, whereas green arrows indicate DPMAS. AST, aspartate transaminase, reference range 13–35 U/L; ALT, alanine aminotransferase, reference range 7–40 U/L; TBil, total bilirubin, reference range 0–21 μmol/L; DBil, direct bilirubin, reference range 0–4 μmol/L. PE, plasma exchange; DPMAS, double plasma molecular adsorption system.

A 47-year-old woman presented with a 10-day history of skin ulceration with serous discharge in the upper inner quadrant of the right breast. Physical examination revealed a firm, non-tender mass measuring approximately 4 cm. Pathology confirmed invasive ductal carcinoma (T4bN0M0, stage IIIB), while no malignant cells were identified in the lymph nodes. Immunohistochemistry showed SOX10 (+), p53 (nonsense mutation), AR (weakly +), ER (−), PR (−), HER2 (−), Ki-67 (~90%+), p63 (−), and GATA-3 (focally +). The patient was diagnosed with triple-negative breast cancer. Baseline laboratory tests, including liver function, coagulation parameters, renal function, and C-reactive protein, were within normal ranges. Serological tests for viral hepatitis including hepatitis B virus DNA, hepatitis C virus RNA, hepatitis E Virus IgM, cytomegalovirus (CMV) DNA, Epstein–Barr virus (EBV) DNA, herpes simplex virus (HSV) IgM, and autoimmune hepatitis antibodies, ferritin, ceruloplasmin, and serum copper were negative. There was no other clear offending drug exposure including concomitant medications, herbal products and supplements et al. The Roussel Uclaf Causality Assessment Method (RUCAM) was calculated to be +8 (5). On July 1, 2025, the patient initiated combined chemotherapy and immunotherapy with nab-paclitaxel (140 mg on days 1, 8, and 15), carboplatin (150 mg on days 1, 8, and 15), and pembrolizumab (200 mg on day 1). The first cycle was well tolerated without significant adverse events. The second cycle was started on July 25, 2025. Three days after treatment initiation on day 1, the patient developed jaundice of the skin and sclera. Laboratory tests revealed markedly elevated liver enzymes and bilirubin (alanine aminotransferase, ALT 97 U/L, aspartate transaminase, AST 168 U/L, gamma-glutamyltransferase, GGT 277 U/L, alkaline phosphatase, ALP 318 U/L, total bilirubin, TBil 136 μmol/L, direct bilirubin, DBil 120 μmol/L), with mild coagulation abnormalities (prothrombin time, PT 13.30 s, prothrombin time activity, PTA 76%, international normalized ratio, INR 1.19). She was admitted to the Department of Hepatology and received hepatoprotective therapy along with empirical meropenem. Abdominal magnetic resonance imaging revealed no evidence of infection, biliary obstruction, or tumor metastasis. Despite treatment, liver enzymes continued to rise rapidly without hepatic encephalopathy, and methylprednisolone therapy was initiated. From August 6 to August 17, intravenous methylprednisolone (40 mg/day) was administered. The patient underwent plasma exchange (PE) four times (August 7–10) and double plasma molecular adsorption system (DPMAS) therapy three times (August 12, 16, and 18). Follow-up tests showed partial improvement in transaminases but persistent hyperbilirubinemia (AST 116 U/L, ALT 102 U/L, GGT 633 U/L, TBil 221.04 μmol/L). Methylprednisolone was then switched to oral administration (16 mg twice daily). On September 2, liver function tests showed AST 70 U/L, ALT 26 U/L, GGT 405 U/L, and TBil 353.18 μmol/L, after which the patient requested discharge. On September 24, 2025, she was readmitted due to severe jaundice. Intravenous methylprednisolone (40 mg/day) was restarted and gradually tapered, followed by oral therapy. Five additional sessions of DPMAS were performed (September 25, 29; October 1, 4, and 8). During treatment, the (1–3)-*β*-D-glucan assay was positive, although chest CT, urinalysis, and urine fungal culture were unremarkable. Prophylactic caspofungin was administered, and the test result turned negative after 10 days. The patient stabilized and was discharged on October 21 with continued steroid tapering. At follow-up on December 15, liver and coagulation functions had significantly improved (Figure 1 and Supplementary Figure 1), and methylprednisolone was discontinued. The patient remained clinically stable through January 28, 2026. Changes in the glucocorticoid regimen are shown in Supplementary Figure 2.

## Discussion

ICIs are monoclonal antibodies targeting immune checkpoint pathways and represent a major advance in oncology (6). However, enhanced immune activation may lead to irAEs, including immune-mediated hepatotoxicity (IMH) (7, 8). Acute hepatitis induced by ICIs is uncommon, with clinical trials of pembrolizumab reporting grade 3–4 hepatotoxicity in <1% of patients. Most cases are mild and self-limiting, whereas severe hepatotoxicity is exceedingly rare (2). The underlying mechanisms remain incompletely understood but are thought to involve dysregulated immune activation, including T-cell–mediated hepatocyte injury, cytokine imbalance, and loss of immune tolerance (7, 9). ICIs have greatly expanded the therapeutic options available for patients with cancer, combination therapy with PD-1/PD-L1 inhibitors and chemotherapy is increasingly used, and its impact on hepatotoxicity risk remains unclear (4), Guo et al. (4) reported that, compared with chemotherapy alone, treatment with PD-1/PD-L1 inhibitors combined with chemotherapy is associated with a higher risk of liver injury of any grade. Current evidence suggests that the association between irAEs and clinical outcomes in patients with cancer remains inconclusive. Some studies have indicated that the development of irAEs may be associated with improved overall survival (OS); however, further research is needed to elucidate this complex relationship (10, 11).

Hepatotoxicity is an important factor limiting the use of anticancer therapies. As the liver plays a central role in the metabolism of numerous drugs and toxins, it is particularly susceptible to drug-induced injury, including that associated with cytotoxic chemotherapy. Moreover, drug-induced liver injury may exhibit cumulative effects, whereby pre-existing hepatic damage can increase the susceptibility to subsequent insults and exacerbate overall liver injury (3). Consistent with the findings of Lasagna et al. (12), the liver injury in this case was likely multifactorial. Chemotherapy may have contributed to the initial hepatic insult, while pembrolizumab may have perpetuated the immune-mediated inflammatory response, ultimately leading to severe liver injury. Moreover, pembrolizumab-induced liver injury has been reported to have a delayed onset (13). ICIs may increase the susceptibility of the liver to chemotherapy-induced injury (14).

Chemotherapy-induced liver injury is relatively uncommon and usually occurs after multiple treatment cycles, typically within 1–4 weeks after chemotherapy (15, 16). According to Justin Floyd et al. (17), approximately 15% of patients receiving carboplatin develop elevations in liver enzymes and/or bilirubin during treatment, however, platinum-based agents rarely cause severe hepatotoxicity, carboplatin may cause mild and transient transaminase elevations but rarely leads to clinically significant hepatotoxicity, with bone marrow suppression as its main dose-limiting toxicity (16, 18). Hruban et al. (18) reported a case of carboplatin-induced liver failure in 1991; however, the patient had a pre-existing history of cirrhosis before receiving treatment. Taxanes are primarily eliminated through hepatic metabolism. Paclitaxel has been associated with elevations in serum transaminases in 7–26% of patients, with occasional mild increases in bilirubin levels. These abnormalities are generally asymptomatic, mild, and self-limiting, rarely requiring dose modification or treatment discontinuation. To date, there have been no reports of clinically significant hepatic dysfunction caused by paclitaxel (16, 19). Before treatment initiation, a liver biopsy was considered. However, the procedure was deferred because the patient presented with coagulopathy and was in critical condition. After clinical stabilization, the patient declined further liver biopsy. Unfortunately, we acknowledge that the exact cause of the hepatotoxicity cannot be definitively determined. It remains unclear whether the liver injury was attributable to pembrolizumab, the chemotherapy agents, or a combination of both. ICI-induced liver injury most commonly presents as a hepatocellular pattern of injury and is typically characterized histologically by panlobular hepatitis with inflammatory infiltrates predominantly composed of lymphocytes and macrophages, sometimes accompanied by portal inflammation. In contrast, the cholestatic pattern is less common (13, 20, 21). Although a liver biopsy was not performed in this case, the pattern of liver function test abnormalities suggested that the patient most likely had mixed-pattern liver injury. However, IMH lacks specific histopathological features, and in the absence of substantial diagnostic uncertainty, liver biopsy was unlikely to influence clinical management.

Refractory severe liver injury associated with ICIs and/or chemotherapy often requires a multimodal therapeutic approach (13). Evidence suggests that ALSS, PE and DPMAS, can reduce systemic inflammation and remove immune mediators, thereby improving outcomes in liver failure (22). PE can replenish deficient coagulation factors, while DPMAS is effective in removing inflammatory mediators and bilirubin without plasma supplementation (23). Most published reports have focused on severe liver injury induced by immune checkpoint inhibitors, suggesting that delayed intervention may lead to liver failure or fatal hepatitis (Table 1). Patients presenting with severe manifestations, such as jaundice and coagulopathy, may require repeated artificial liver support and prolonged corticosteroid therapy (24). However, treatment responses may vary (Table 1). Riveiro-Barciela et al. (25) reported a case of a melanoma patient who developed acute liver failure after immunotherapy and survived following treatment with immunosuppressive therapy combined with plasma exchange (PE). They suggested that PE is the preferred treatment modality for certain immune-mediated diseases, such as Guillain–Barré syndrome. PE has been shown to increase the number of regulatory T cells (Tregs) and may also accelerate the clearance of ipilimumab.

**Table 1:** Patients with severe hepatitis induced by immunotherapy or chemotherapy.

Author	Year	Age/sex	Antitumor therapy (course)	Biochemistry	Treatment (course)	Outcome
Ahmed et al.	2015	50/F	Ipilimumab (unknown)	AST 7280 U/L ALT 4700 U/L	Corticosteroid +ATG + MMF (6 weeks)	Survival
Simonelli et al.	2016	68/M	Nivolumab (4 cycles)	AST 426 U/L ALT 1102 U/L GGT 258 U/L TBil 25.0 μmol/L	Corticosteroid (3 weeks)	Survival
Hajj et al.	2016	72/F	Cisplatin + 5-fluorouracil (5-FU) (1 cycle)	AST 226 U/L ALT 126 U/L ALP 51 U/L GGT 106 U/L TBil 430 μmol/L	Unknown	Death
Barciela et al.	2018	76/F	Ipilimumab (2 cycles)	AST 4470 IU/mL ALT 2530 IU/mL TBil 179.6 μmol/L PTA 24%	PE + Corticosteroid + MMF (1 month)	Survival
Bhave et al.	2018	47/M	Nivolumab + Ipilimumab (4 cycles)	AST 881 U/L ALT 2259 U/L GGT 121 U/L INR 3.6	Corticosteroid + MMF (12 days)	Death
Inamori et al.	2019	80/F	Nivolumab + Sunitinib (4 cycles)	AST 873 U/L ALT 317 U/L TBil 202.1 μmol/L	Corticosteroid (10 days)	Death
Yaegash et al.	2019	59/F	Cisplatin + 5-fluorouracil (5-FU) (1 cycle)	AST 1193 U/L ALT 778 U/L	Corticosteroid (4 days)	Survival
Calderon et al.	2021	56/F	Pembrolizumab (3 cycles)	AST 650 U/L ALT 826 U/L GGT 728 U/L TBil 5.5 μmol/L INR 1.0	Corticosteroid (1 month)	Survival
Otsuka et al.	2023	58/M	Pembrolizumab + cisplatin + 5-fluorouracil (5 cycles)	AST > 500 U/L ALT > 3,000 U/L	Corticosteroid + MMF + Tacrolimus (8 months)	Survival
Renault et al.	2023	64/M	Atezolizumab + Tiragolumab or placebo (4 cycles)	AST 948 U/L ALT 948 U/L TBil > 300 μmol/L PTA 28%	PE + Corticosteroid + MMF (unknown)	Survival

M, male; F, female; AST, aspartate transaminase; ALT, alanine aminotransferase; GGT, gamma-glutamyltransferase; ALP, alkaline phosphatase; TBil, total bilirubin, PTA, prothrombin time activity; INR, international normalizing ratio; PE, plasma exchange; ATG, antithymocyte globulin; MMF, mycophenolate mofetil.

## Conclusion

This case highlights the need for close monitoring of liver function during combined chemo-immunotherapy. Notably, the patient exhibited prolonged hepatocellular injury even after treatment discontinuation, although disease severity gradually improved. Severe liver dysfunction caused by combined therapy, particularly early onset hepatitis, remains extremely rare and warrants further investigation to better define its risk factors and optimal management strategies.

## Data Availability

The original contributions presented in the study are included in the article/Supplementary material, further inquiries can be directed to the corresponding author.
