# Supplementary material for: Successful management of severe hepatotoxicity induced by combined chemotherapy and immune checkpoint inhibitor: a case report
Source: Front Med (Lausanne). 2026 Aug 12;13:1886401. doi: 10.3389/fmed.2026.1886401 (PMC13507675; doi:10.3389/fmed.2026.1886401)
Supplement: Supplementary file 1 [file Table_1.DOCX]

FIGURE 1

Laboratory data of this patient, obtained during hospitalization. **(A)** PT; **(B)** INR. PT, prothrombin time, reference range, 9.4-12.5; INR, international normalizing ratio, reference range, 0.8-1.2.

FIGURE 2

The course of corticosteroid administration. Red arrows indicate PE, whereas green arrows indicate DPMAS. MP, methylprednisolone; i.v., intravenously; PO, by mouth orally; q.d., once daily; BD, twice daily; PE, plasma exchange; DPMAS, double plasma molecular adsorption system.
